# The Physiological and Biochemical Responses of a Medicinal Plant (*Salvia miltiorrhiza* L.) to Stress Caused by Various Concentrations of NaCl

**DOI:** 10.1371/journal.pone.0089624

**Published:** 2014-02-25

**Authors:** Zhao Gengmao, Shi Quanmei, Han Yu, Li Shihui, Wang Changhai

**Affiliations:** College of Resources and Environmental Sciences, Nanjing Agricultural University, Nanjing, Jiangsu Province, P.R. China; DOE Pacific Northwest National Laboratory, United States of America

## Abstract

*Salvia miltiorrhiza*, which is commonly known as Danshen, is a traditional Chinese herbal medicine. To illustrate its physiological and biochemical responses to salt stress and to evaluate the feasibility of cultivating this plant in saline coastal soils, a factorial experiment under hydroponic conditions was arranged on the basis of a completely randomised design with three replications. Five salinity treatments (0, 25, 50, 75 and 100 mM NaCl) were employed in this experiment. The results showed that salinity treatments of <100 mM NaCl did not affect the growth of *Salvia miltiorrhiza* in a morphological sense, but significantly inhibit the accumulation of dry matter. Salinity treatments significantly decreased the Chl-b content but caused a negligible change in the Chl-a content, leading to a conspicuous overall decrease in the T-Chl content. The Na^+^ content significantly increased with increasing hydroponic salinity but the K^+^ and Ca^2+^ contents were reversed, indicating that a high level of external Na^+^ resulted in a decrease in both K^+^ and Ca^2+^ concentrations in the organs of *Salvia miltiorrhiza*. Salt stress significantly decreased the superoxide dismutase (SOD) activity of *Salvia miltiorrhiza* leaves in comparison with that of the control. On the contrary, the catalase (CAT) activity in the leaves markedly increased with the increasing salinity of the hydroponic solution. Moreover, the soluble sugar and protein contents in *Salvia miltiorrhiza* leaves dramatically increased with the increasing salinity of the hydroponic solution. These results suggested that antioxidant enzymes and osmolytes are partially involved in the adaptive response to salt stress in *Salvia miltiorrhiza*, thereby maintaining better plant growth under saline conditions.

## Introduction

Salt stress limits agricultural production throughout the world and is becoming an increasingly global problem that affects approximately 20% of global irrigated land [1]. In China, saline soil is mostly located in the north-western inland regions, where limited rainfall, high evapotranspiration, and possible poor soil water management lead to soil salinization. In addition to inland saline soils, there are nearly 2,000,000 hectares of saline soils on the eastern coastline of China. Coastal saline soils have recently been eagerly considered by local governments for developing valuable salt-tolerant plants, such as Jerusalem artichokes [2], sunflowers [3] and some medicinal plants [4], [5], thus attracting high investigator interest in these plants. High salinity causes various cooperative events that negatively impact agricultural production, such as delays in plant growth and development [6]; thus, investigations on the salt tolerance mechanisms of these plants are very important.

Salt stress can reportedly result in oxidative stress through increased reactive oxygen species (ROS), such as the superoxide radical (O_2_), hydrogen peroxide (H_2_O_2_), and the hydroxyl radical (OH) [7]. These ROS may be signals for inducing ROS scavengers and other protective mechanisms, and they could be damaging agents that contribute to stress injuries in plants [8]. To mitigate ROS-mediated oxidative damage, plants have developed a complex antioxidative defence system that includes ionic and osmotic homeostasis and antioxidative enzymes. Ionic homeostasis plays an important role in the physiology of all living cells, and the regulation of ion fluxes is important for ensuring that the concentration of essential ions is greater and the toxic ion concentration is below the range that can create an ion imbalance [9], [10]. Some plants also have the potential to increase cellular concentrations of osmotically compatible solutes [11]–[14]. These compatible solutes help to maintain ion homeostasis and water relations, and they alleviate the negative effects of high ion concentrations on the enzymes, stabilising proteins, protein complexes, membranes and cellular function under stress conditions [15]. Moreover, salinity-induced oxidative stress could be ameliorated through the action of antioxidative enzymes such as superoxide dismutase (SOD), peroxidase (POD), ascorbate peroxidase (APX), and catalase (CAT).

Medicinal plants are among the major important crops [16]. Although the effects of salt stress on traditional crops have been extensively investigated, there is a lack of information in the case of medicinal plants [17]. *Salvia miltiorrhiza*, or danshen, is an annual sage primarily found in China and neighbouring countries, and its crude drug (dried root) and preparations are currently used in China to treat patients suffering from heart attacks, angina pectoris, strokes and other conditions. The physiological and biochemical responses of medicinal plants (*Salvia miltiorrhiza* L.) to salt stress have not been reported in the literature. In this paper, investigations of ionic and osmotic homeostasis and antioxidative enzymes could provide comprehensive insights into the response mechanisms induced by salt stress in *Salvia miltiorrhiza* plants. Therefore, we investigated the effects of salt stress on plant growth characteristics, photosynthesis, and ion and osmotic homeostasis, in addition to antioxidative enzymes in *Salvia miltiorrhiza* leaves.

## Materials and Methods

### Experimental layout

*Salvia miltiorrhiza* seeds (*Salvia miltiorrhiza* Bunge. cv. Sativa) were collected from the Zhongjiang region, Shichuan Province in September 2013, and they were naturally preserved in the Marine Environment and Ecology Lab of Nanjing Agricultural University until the experiment started on March 11, 2013. The seeds were surface-sterilised with 2.5% sodium hypochlorite solution and rinsed with distilled water. They were then transferred to sterile moist filter paper after swelling in distilled water at 25°C for 12 h. The seeds were placed in a glass Petri dish for germination at 28°C for 12 h in the daylight and 22°C for 12 h in the dark. The seeds germinated 72 h later, and uniformly germinated seeds were selected and cultivated in holes within Styrofoam boards filled with organic media made of humus, perlite and vermiculite at a ratio of 4∶1∶1.

The seedlings were grown under the following cultivation conditions: 28°C/22°C day and night temperatures and a relative humidity of 70–80% in a semi-controlled plant greenhouse in the Pailou base of Nanjing Agricultural University. When the 5th new leaf emerged, the seedlings were transferred to 1/2 Hoagland nutrient solution for further cultivation. The nutrient solution was aerated continuously and replaced every two days. For the salt treatments, soluble NaCl was added to the culture medium until the salt concentrations reached 25, 50, 75 and 100 mM. The hydroponic experiment was arranged as a factorial setup on the basis of a completely randomised design with three replications. *Salvia miltiorrhiza* seedlings were exposed to salt stress by adding NaCl to the culture medium when plants were 3- weeks old on March 31 and harvested on April 30. While harvesting, plant samples were collected for analysing the physiological and biochemical parameters of *Salvia miltiorrhiza*, such as photosynthetic pigments, antioxidant enzymes and compatible solutes.

### Physiological and biochemical measurements

#### Assay for plant growth characteristics and ion concentrations in plant organs

The growth characteristics of *Salvia miltiorrhiza* such as the plant height, root length and plant biomass were measured to according to Lu [18]. Plant tissue samples were dried at 65°C for 6 d, weighed, and ground in a Wiley mill to pass through a 40 mesh screen. Subsamples were digested by using a hydrogen peroxide (H_2_O_2_)/nitric acid (HNO_3_) digest according to Huang and Schulte [19] and analysed by inductively coupled emission spectroscopy at the Nanjing Agricultural University Life Science Analysis Laboratory (ICP-OES, IRIS plasma spectrophotometer).

#### Measuring photosynthetic pigments in *Salvia miltiorrhiza* leaves

The leaf tissues of *Salvia miltiorrhiza* were ground in liquid nitrogen, and 1 ml of 100% acetone was added to extract the chlorophyll. After vigorously vortexing at 4°C for 1 h in the dark, cell debris was removed by centrifugation at 15,000×g at 4°C for 15 min. The concentrations of chlorophyll a (Chl a), chlorophyll b (Chl b) and total chlorophyll (T-Chl) were calculated by using the Lichtenthaler equations as follow: Chl a = 11.24×A661.6−2.04×A644.8; Chl b = 21.13×A644.8−4.19×A661.6 and tChl = 18.09×A644.8+ 7.05×A661.6 [20].

#### Antioxidant enzyme assays in *Salvia miltiorrhiza* leaves

To determine antioxidant enzyme activities, 0.5 g of *Salvia miltiorrhiza* leaves were homogenised with ice-cold 50 mM KPi (pH 7.0) containing 0.1 mM EDTA, 1% (w/v) polyvinylpyrrolidone (PVP) and 0.5% (v/v) Triton X-100 at 4°C. The homogenate was filtered through four layers of cheesecloth and centrifuged at 14,000×g for 15 min at 4°C. The supernatant was collected to determine the antioxidant enzyme activities and stored at −80°C for additional analyses [21]. The CAT activity was assayed by measuring the initial H_2_O_2_-scavenging rate. The extinction coefficient for H_2_O_2_ at 240 nm was 40 mM^−1^ cm^−1^ [22]. The SOD activity was assayed according to the method described by Kwak et al. by using pyrogallol as a substrate [23]. The molar extinction coefficient of purpurogallin is 2.47 mM^−1^ cm^−1^.

#### Assaying for compatible solutes in *Salvia miltiorrhiza* leaves

To measure the soluble sugar content, 0.5 g of dry leaves was homogenised with 5 ml of 95% ethanol. One-tenth of one ml of alcoholic extract was preserved in the refrigerator and mixed with 3 ml of anthrone (150 mg anthrone and 100 ml of 72% sulphuric acid, w/w). The samples were placed in a boiling water bath for 10 minutes. The light absorption of the samples was estimated at 625 nm by using a spectrophotometer. The soluble sugar contents were determined by using a glucose standard and expressed as mg g^−1^ DW of leaves.

Harvested leaf samples were immediately frozen in liquid nitrogen and stored at −80°C until use. From each sample, 250 mg was extracted in 0.8 ml of Tris-boric buffer (0.09 M Tris, 0.08 M boric acid, and 0.93 g/l of Na_2_EDTA) and 0.8 ml of 40% sucrose (w/v), and then the extraction was centrifuged at 10000 g for 10 min. The supernatant was mixed with an equal volume of Laemmli solution (1 M Tris (pH = 8.8), 0.4 g of SDS, 0.8 g of glycerol, and 0.9 ml 2-ME (mercaptoethanol) in 10 ml of dd H_2_O, heated in boiling water for 5 min, and frozen until use. The total soluble protein content was determined according to the Lowry et al. method [24] with bovine serum albumin as the standard.

### Statistical analysis

The mean values of all parameters were taken from the measurements of three replicates, and the standard error of the means was calculated. One-way ANOVA was applied to determine the significance of the results between different treatments and Tukey's multiple range tests (P<0.05) were then performed. All statistical analyses were performed by SPSS v.13 for Windows.

## Results

### Salt stress effects on the growth of *Salvia miltiorrhiza* seedlings

Salt stress exerted differential effects on the growth parameters, such as the plant height, root length and plant biomass of *Salvia miltiorrhiza* (Table 1).

**Table 1:** The effects of various NaCl concentrations on the plant height, root length, fresh weight and dry weight of *Salvia miltiorrhiza* seedlings.

Treatments	Plant growth characteristics			
	Plant height	Root length	Fresh weight	Dry weight
	(cm)	(cm)	(g/plant)	(g/plant)
0 mM NaCl	30.5±4.4a	11.4±2.0a	21.59±5.24a	3.42±0.56a
25 mM NaCl	28.7±4.7a	11.3±2.1a	16.28±6.18ab	2.12±0.37b
50 mM NaCl	26.7±5.5a	11.2±2.5a	16.06±5.21ab	2.08±0.52b
75 mM NaCl	26.0±4.5a	11.0±2.5a	10.68±4.34b	1.83±0.45b
100 mM NaCl	26.3±3.3a	10.9±1.6a	9.77±3.05b	1.76±0.51b

The seedlings were cultivated in 1/2 Hoagland nutrient solution for 3 weeks and were later exposed to salt stress by adding NaCl to concentrations of 25, 50, 75 and 100 mM of the hydroponic solution for 30 days. Non-treated plants were used as controls (0 mM NaCl). Each value represents the mean of three replicates. Treatments with the same letters are not statistically different (P≥0.05).

When *Salvia miltiorrhiza* was exposed to salt stress conditions for 30 days, high salinity treatments at 75 and 100 mM NaCl significantly (P<0.05) decreased the plant fresh and dry weights compared with that of CK, and the plant height and root length remained virtually unchanged among all salt treatments. In addition, the low salinity 25 and 50 mM NaCl stresses did not affect the plant fresh weight but markedly decreased the plant dry weight. These results suggested that salt stress of <100 mM NaCl did not affect *Salvia miltiorrhiza* growth on a morphological basis, but could significantly inhibit the accumulation of dry matter.

### Salt stress effects on photosynthetic pigments in *Salvia miltiorrhiza* leaves

Photosynthesis in plants is dependent on capturing light energy in the pigment chlorophyll, particularly chlorophyll a (Chl-a) and chlorophyll b (Chl-b).

**Figure 1:**
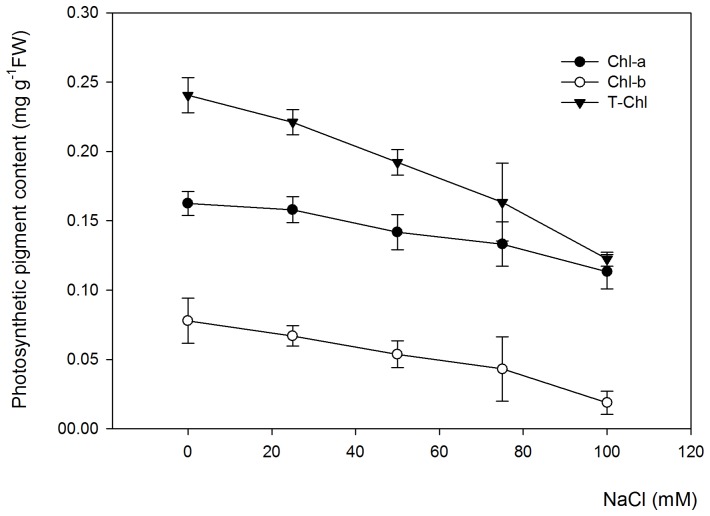
The effects of various NaCl concentrations on the photosynthetic pigments of *Salvia miltiorrhiza* leaves. The photosynthetic pigments measured in the experiment include chlorophyll a (Chl-a), chlorophyll b (Chl-b) and total chlorophyll contents (T-Chl). *Salvia miltiorrhiza* seedlings were cultivated in 1/2 Hoagland nutrient solution for 3 weeks and were later exposed to salt stress by adding NaCl up to concentration of 25, 50, 75 and 100 mM of the hydroponic solution for 30 days. Non-treated plants were used as controls (0 mM NaCl). Error bars represent the standard errors (SE) of the means.

Under salt stress conditions, *Salvia miltiorrhiza* showed a significant (P<0.05) decrease in Chl-b content but a negligible change in Chl-a content, leading to a conspicuous overall decrease in the T-Chl content(Figure 1). In addition, the Chl-b content was decreased with increasing hydroponic salinity, suggesting that salt stress markedly affected the Chl-b biosynthesis of *Salvia miltiorrhiza* leaves.

### Salt stress effects on the ionic homeostasis of *Salvia miltiorrhiza* seedlings

Salinity accompanied by high salt concentrations would induce an ionic imbalance in plants. Plant tolerance to salinity stress primarily depends on the low Na^+^ and the maintenance of high nutrient concentrations, especially K^+^ and Ca^2+^ homeostasis. K^+^/Na^+^ and Ca^2+^/Na^+^ ratios are important parameters that reflect the capacity for ionic homeostasis in plants. Under salt-stressed conditions, changes in the ion concentrations of *Salvia miltiorrhiza* seedlings were measured and shown in Table 2.

**Table 2:** The effects of various NaCl concentrations on the ion content and selective absorption of *Salvia miltiorrhiza* seedlings.

Treatments	Ions contents (µmol g^−1^ DW)				
	Na^+^	K^+^	Ca^2+^	K^+^/Na^+^ ratio	Ca^2+^/Na^+^ ratio
0 mM NaCl	16.1±0.4e	898.7±8.7a	275.8±3.0a	55.9	17.1
25 mM NaCl	167.8±1.7d	769.7±12.1b	275.3±10.3a	4.6	1.6
50 mM NaCl	393.5±23.0c	415.1±9.0c	251.0±1.0b	1.1	0.6
75 mM NaCl	464.8±11.3b	243.8±3.8d	226.0±2.0c	0.5	0.5
100 mM NaCl	502.2±23.0a	224.6±5.6e	221.5±10.5c	0.4	0.4

The seedlings were cultivated in 1/2 Hoagland nutrient solution for 3 weeks and were later exposed to salt stress by adding NaCl to concentrations of 25, 50, 75 and 100 mM of the hydroponic solution for 30 days. Non-treated plants were used as controls (0 mM NaCl). Each value represents the mean of three replications. Treatments with the same letters are not statistically different (P≥0.05).

When *Salvia miltiorrhiza* was exposed to salt stress conditions for 30 days, the Na^+^ content in salt-stressed treatments was significantly (P<0.05) increased with the increasing salinity of the hydroponic solution, but the K^+^ content was reversed. For the Ca^2+^ content, the highest value was obtained in CK, followed by 25, 50, 75 and 100 mM NaCl treatments. In addition, the K^+^/Na^+^ and Ca^2+^/Na^+^ ratios also decreased with the increasing salinity of the hydroponic solution. These results indicated that a high level of external Na^+^ resulted in a decrease in both the K^+^ and Ca^2+^ concentrations in *Salvia miltiorrhiza*.

### Salt stress effects on the antioxidant enzyme activities of *Salvia miltiorrhiza* leaves

**Figure 2:**
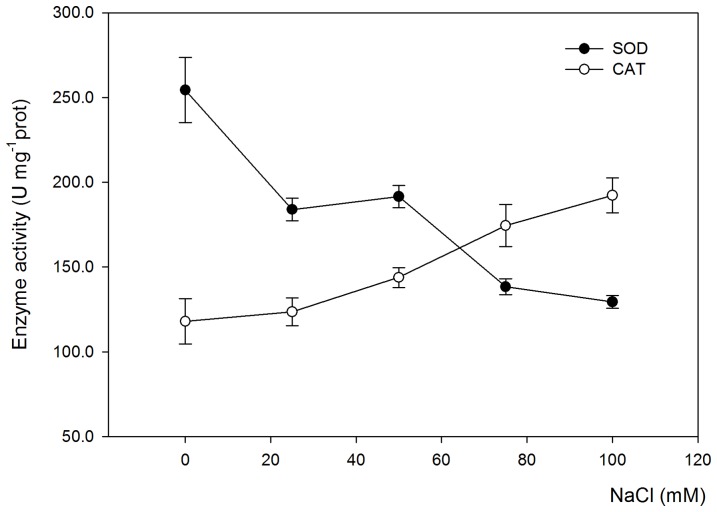
The effects of various NaCl concentrations on the enzyme activities of *Salvia miltiorrhiza* leaves. The enzyme activities measured in this experiment include superoxide dismutase (SOD) and catalase (CAT) activities. *Salvia miltiorrhiza* seedlings were cultivated in 1/2 Hoagland nutrient solution for 3 weeks and were later exposed to salt stress by adding NaCl to concentrations of 25, 50, 75 and 100 mM of the hydroponic solution for 30 days. Non-treated plants were used as controls (0 mM NaCl). Error bars represent the standard errors (SE) of the means.

Plants seem to activate a complex antioxidative defence system in response to salt stress that is displayed via the increase or decrease of enzymatic activities. In this experiment, the SOD and CAT activities of *Salvia miltiorrhiza* leaves were measured under NaCl-stressed conditions (Figure 2). The results showed that salt stress significantly (P<0.05) decreased the SOD activity of *Salvia miltiorrhiza* leaves in comparison with that of the control, indicating that salt stress adversely affected the SOD activity, and thus resulting in the reduction in the scavenging capacity of O_2_^−^. Moreover, no conspicuous differences in the SOD activity of *Salvia miltiorrhiza* leaves were observed between 25 and 50 mM NaCl treatments, and a similar phenomenon was found between 75 and 100 mM NaCl treatments. On the contrary, the CAT activity of *Salvia miltiorrhiza* leaves markedly increased with the increasing salinity of the hydroponic solution, suggesting that the scavenging capacity of H_2_O_2_ was increased to a great extent by salt stress.

### Salt stress effects on the osmolyte concentration of *Salvia miltiorrhiza* leaves

**Figure 3:**
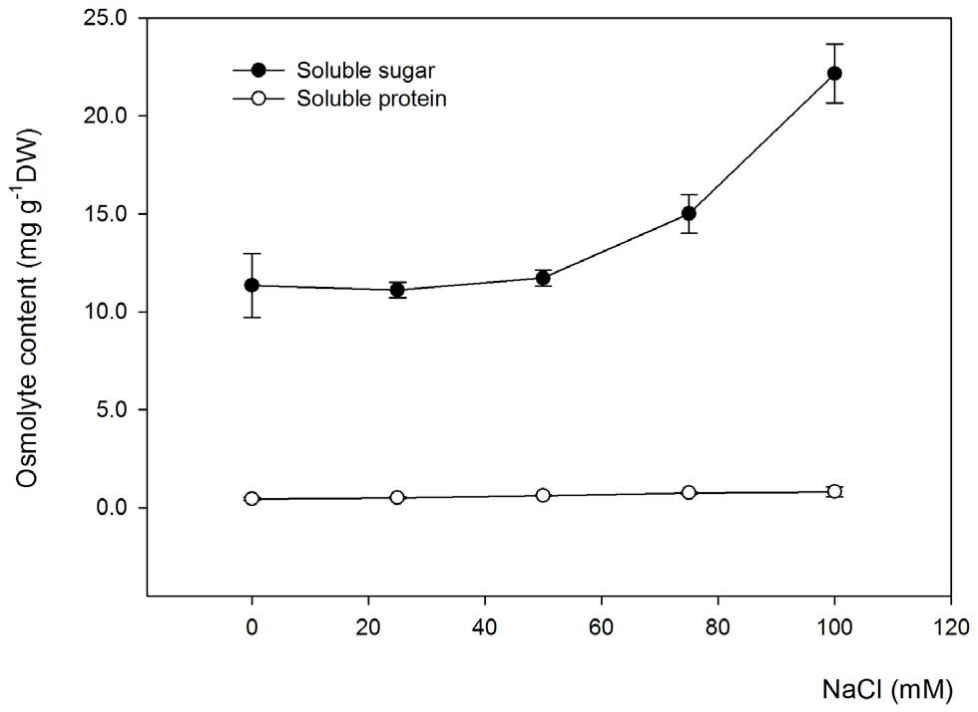
The effects of various NaCl concentrations on the osmolyte contents of *Salvia miltiorrhiza* leaves. The osmolyte contents measured in the experiment include the soluble protein and soluble sugar contents. *Salvia miltiorrhiza* seedlings were cultivated in 1/2 Hoagland nutrient solution for 3 weeks and were later exposed to salt stress by adding NaCl up to 25, 50, 75 and 100 mM of the hydroponic solution for 30 days. Non-treated plants were used as controls (0 mM NaCl). Error bars represent the standard errors (SE) of the means.

Soluble sugar and protein, which are the primary osmolytes in plants, play key roles in osmotic adjustment under salt-stressed conditions. When *Salvia miltiorrhiza* underwent salt stress, the soluble sugar and protein contents in the leaves increased as shown in Figure 3. Also, the soluble sugar content of *Salvia miltiorrhiza* leaves dramatically increased with the increasing salinity of the hydroponic solution.

In addition, the salt stress from various NaCl concentrations significantly (P<0.05) increased the soluble protein content of *Salvia miltiorrhiza* leaves compared with the control. These results indicated that *Salvia miltiorrhiza* partly adapted to saline environments or weakened oxidative stress induced by salt applications through an increase of soluble sugar and protein in the leaves.

## Discussion

### *Salvia miltiorrhiza* seedling growth in response to salt stress

One of the initial effects of salt stress on plants is reduced growth characteristics. According to Munns [25], plant responses to salt stress can be divided into two phases. The first phase of growth reduction is a quicker process caused by an osmotic effect. The second phase, however, is a much slower process caused by salt accumulation in leaves, leading to salt toxicity in the plants. Our results showed that high salinity significantly decreased plant fresh and dry weights when compared with those of the control (Table 1). These findings were similar to earlier data in different crop species e.g., wheat [26], soybean [27], canola [28] and some halophytic plants [29]. Additionally, the plant height and root length of *Salvia miltiorrhiza* remained virtually unchanged among all the salt-treated treatments, suggesting the growth of the plant was not affected on a morphological basis. However, many investigators have reported the adverse effects of salt stress on plant height and root length [30], [27]. This finding might have occurred because the tested *Salvia miltiorrhiza* had higher salt tolerance and could bear more than 100 mM in NaCl stress.

### Photosynthesis in *Salvia miltiorrhiza* leaves in response to salt stress

Photosynthetic pigments such as chlorophyll ‘a’ and ‘b’ play key roles in photosynthesis [31]. A reduction in photosynthesis caused by salt stress is partly ascribed to reduced chlorophyll contents [32]. Our results showed a significant decrease in the Chl-b content but a negligible change in the Chl-a content, leading to a conspicuous overall decrease in the T-Chl content (Figure 1). The adverse effect of photosynthesis under salt-stressed conditions was identified earlier in different crop species, e.g., Sultana vines [33], cowpea [34], cotton [35] and wheat [36]. However, there are many reports showing little or no changes or even stimulation in the photosynthesis capacity of plants under low salinity stress [37], [38]. In fact, the effect of salinity on photosynthesis depends on the salt concentration in addition to the plant species or genotypes.

### Ion contents of *Salvia miltiorrhiza* seedlings in response to salt stress

Ion toxicity is the most important factor in the considerable reduction of plant vegetative growth, thus affecting the desirable yield achieved during harvest [13]. Plant survival under saline conditions primarily depends on the ion homeostasis. Ion flux regulation is important for ensuring that the concentration of essential ions is greater and the toxic ions are below the range that can create an ionic imbalance [9], [10]. Our results showed that the Na^+^ content in salt-treated *Salvia miltiorrhiza* was significantly increased with the increasing salinity of the hydroponics but the K^+^ content was reversed, implying that a high level of external Na^+^ caused a decrease in the K^+^ concentrations in *Salvia miltiorrhiza* tissues (Table 2). This finding was consistent with results from other cultivars [39]–[41]. Moreover, the K^+^/Na^+^ and Ca^2+^/Na^+^ ratios of *Salvia miltiorrhiza* were also decreased with the increasing salinity of the hydroponic solution. These findings were comparable with observations in other crops, for example safflower studied by Patil et al. [42], in which the average K^+^/Na^+^ ratio of the total plant decreased with the increased NaCl concentrations, indicating that there is no selective absorption mechanism in these plants. Thus, a low K^+^/Na^+^ ratio may result in metabolic disorders such as reduced protein syn­thesis and enzyme activities [43] and an increase in the membrane permeability [44].

### The antioxidant enzyme activities of *Salvia miltiorrhiza* leaves in response to salt stress

Scavenging ROS through the increased activation of antioxidant enzymes can improve salt tolerance [45]. Salt stress significantly decreased the SOD activity of *Salvia miltiorrhiza* leaves in comparison with that of the control, indicating that salt stress adversely affected the SOD activity. On the contrary, the CAT activity of *Salvia miltiorrhiza* leaves markedly increased with the increasing salinity of the hydroponic solution (Figure 2). These findings were comparable with the observation by Perveen et al. [46], in which salinity stress of 150 mM NaCl significantly decreased the SOD activity, and it increased that of CAT in wheat. However, most of the literature showed that the plant's SOD activity increased under salt stress conditions [47]–[49]. Increased SOD activity enables plants to resist the potential oxidative damage caused by NaCl salinity exposure [50], [51], but other antioxidant enzymes such as CAT, APX and GR may play vital roles in the salt tolerance of various species or varieties.

### Osmolyte contents of *Salvia miltiorrhiza* leaves in response to salt stress

In salinity stress, plants escape from dehydration by reducing their osmotic potential and by adjusting with osmolytes, which assist in the transport, accumulation and compartmentalisation of organic solutes. Our results showed that the soluble sugar and protein contents in *Salvia miltiorrhiza* leaves dramatically increased with the increasing salinity of the hydroponic solution, indicating that osmolytes, such as soluble sugar and proteins, play key roles in osmotic adjustment under salt-stressed conditions (Figure 3). Salt stress has been widely reported to result in the accumulation of organic osmolytes and compatible solutes, such as soluble sugars, soluble proteins, and proline, to maintain cell turgor and regulate water acquisition [52]–[55], [11]. Therefore, the accumulation of soluble sugars and proteins helps to maintain the ionic homeostasis in most plants including the medicinal plant *Salvia miltiorrhiza*.

It is generally agreed that most plants are salt-sensitive during the seedling stage, but after the seedling stage, they become increasingly tolerant as growth proceeds through the vegetative and reproductive stages. In this paper, we examined the physiological and biochemical bases of salt tolerance during the seedling stage of Salvia miltiorrhiza; however, the important mechanisms associated with tolerance at the vegetative or reproductive stages of growth will require further study to fully characterize how life cycle interacts with salt tolerance for this importance plant.
